# Health technology assessment capacity to support Zambia’s health benefits package reform policy

**DOI:** 10.1017/S0266462325000030

**Published:** 2025-02-28

**Authors:** Warren Mukelabai Simangolwa, Kaymarlin Govender, Josue Mbonigaba

**Affiliations:** 1 Health Economics and HIV/AIDS Research Division, University of KwaZulu Natal, South Africa; 2 College of Law and Management Sciences, University of KwaZulu Natal, South Africa; 3 Centre for Health Economics Financing and Technology Assessment, Patient and Citizen Involvement in Health, Lusaka, Zambia

**Keywords:** Health technology assessment, health benefits package, clinical effectiveness, health economics, capacity

## Abstract

**Background:**

The need for more local technical capacity in Health Technology Assessment (HTA) is a leading challenge to its use in low- and middle-income countries. Zambia has been considering using HTA to support its universal health coverage initiative, which includes health benefits package design and implementation. This study assesses the local HTA capacity for the steering committee tasked with supporting the design and implementation of the national health benefits package in Zambia.

**Methods:**

The study applied a cross-sectional web-based survey design and the consensus-based Checklist for Reporting of Survey Studies. Data were collected from the steering committee of the benefits package working group, tasked with leading the design process of the health benefits package using the Instrument for the Assessment of Skills to Conduct a Health Technology Assessment tool.

**Results:**

The majority of respondents had not served on a selection and reimbursement committee. Clinical effectiveness skills in structuring a search strategy, handling missing data, conducting qualitative evidence synthesis, and grading the certainty of evidence were low. Skills for leadership, networking, conflict management, and project coordination, public and patient involvement were mid-level to low. Most of the respondents were aware of ethical issues with health technologies. Health economics skills in economic evaluations and decision analytic modeling, equity and health system efficiency measurement, budget impact analysis, and quality of life were identified for capacity strengthening.

**Conclusion:**

Available technical capacities to revise and implement the national benefits package were lower in health economics, synthesis for clinical effectiveness evidence, ethics, patient and public involvement, and soft skills, in that order.

## Introduction

Many low- and middle-income countries (LMICs) aspire to use Health Technology Assessment (HTA) to inform evidence synthesis, pricing, reimbursement and purchasing, regulatory and formulary, priority-setting, efficiency analysis, and universal health coverage decisions (1–4). However, advancements in these practical applications in LMICs have been impeded by limited institutionalization of HTA, funding, data availability, coordination and linkages to decision-making, methodological guidelines, and networking (1;3;5–7). Developing local HTA technical capacity is a leading recommendation to advance its desired use (5;7–9). Some countries have instituted on-the-job training, short courses, and university programs on HTA to localize these capacities (2;9–11) based on project management, research, and economic evaluation needs assessments (2;10).

Conducting and applying HTA requires stakeholders to have diverse competencies, such as health economics, project and research leadership, stakeholder engagement, clinical effectiveness research, data synthesis, ethical considerations, consensus building, and involving patients and the public in decision-making (12–14). Key steps in HTA include establishing a committee, defining decision criteria, selecting health technologies, scoping, assessment, appraisal, communicating HTA results, managing appeal processes, and ongoing monitoring and evaluation (15). During the committee establishment, stakeholder mapping and engagement skills are necessary. Conversely, preference elicitation and consensus-building skills are essential during the decision criteria phase. In horizon scanning for technology selection, expertise in information synthesis and networking is crucial, while scoping focuses on research capacity to specify HTA objectives. The assessment phase demands skills in evidence collection, synthesis, and reporting, including clinical effectiveness and economic evaluation analysis, along with undertaking systematic or scoping reviews (16). The ability to interpret scientific research and build consensus is crucial during appraisal. Effective communication and appeal processes require skills in media relations, conflict management, and interpersonal interactions. Finally, the monitoring and evaluation phase calls for skills in impact assessment.

In sub-Saharan Africa, HTA capacity reviews in Tanzania, Ghana, Malawi, Kenya, and South Africa have identified local challenges in producing and using HTA data (10;11;17–19). In Tanzania, a capacity review of the National Medicines and Therapeutic Committee identified skills gaps in critically appraising literature and activity-based costing (10). A skills review for HTA producers in Ghana showed a need for lead roles by local researchers in collaborations for HTA production (17). Health technology funders, donors, and the private sector influenced HTA research priorities and decision-making in Malawi (18). Although an HTA technical working group has been established in Kenya, the technical skills and funding required to conduct HTA have remained low (11). In South Africa, there are concerns that the fragmented and limited coordination of HTA could derail its use to support priority-setting for the newly established National Health Insurance (19).

Zambia strives to achieve universal health coverage to ensure all its citizens receive health services without financial hardship (20). It considers HTA an essential policy instrument to inform the choice of services that should be covered to realize universal health coverage (21). Furthermore, the government of Zambia intends to institutionalize HTA to support formal priority-setting, strategic purchasing, and quality improvement (21;22). However, the use of HTA is limited by the absence of a dedicated governing entity, attrition of research capacity, limited budgetary allocation for research, and externalization of the priority-setting process to donors (13;22). This is coupled with limited formal training opportunities for health economics in local institutions (23).

In a recent initiative, the Ministry of Health launched a five-year roadmap to revise the 2012 National Health Care Package. The roadmap proposed using HTA as part of a broader health allocative efficiency process to improve value-for-money approaches (20). The National Health Care Package is a comprehensive benefits package encompassing primary and hospital-level care. The revision process’s capacity-building objective advocates localizing capacities to guarantee the availability of human resources for future revisions and explore networking opportunities for applying practical skills (20). The technical capacity needed to implement the revision roadmap requires skills in HTA, including costing, evidence collection and synthesis, economic evaluation, project management and communication, stakeholder engagement and decision-making, and patient, political and public involvement (20). This study determines the availability of stakeholder HTA skills needed to support the health benefits package design and implementation process and identifies future HTA capacity development needs in Zambia.

## Methods

### Study design

The study used a cross-sectional web-based survey design. Its conduct and reporting followed the 19 items of the Consensus-Based Checklist for Reporting of Survey Studies (24), which increases trustworthiness, transparency, and robustness in conducting survey-based research (24). The completed checklist is in the Supplementary File 1.

### Data collection methods

The study used the Instrument for the Assessment of Skills to Conduct a HTA tool (14). The tool contains four sections with a total of 49 questions. The general information section solicits information on professional and HTA experience. In contrast, the core skills section assesses stakeholder capacity regarding clinical effectiveness, public involvement, ethics and health economics. Capacities in management, HTA governance, and communication are evaluated in the soft skills section. In the future needs section, stakeholders highlight the skills needed for their capacity development in HTA. The tool has been adapted for capacity assessments in Ghana and Tanzania (10;14).

### Sample characteristics

The Zambia Ministry of Health has established a technical working group to oversee the revision of the National Health Care Package. This working group comprises stakeholders likely to promote HTA and produce HTA-related evidence within the Ministry of Health, other ministries within the government, cooperating partners, the private sector, regulatory institutions, public and patient representatives, civil society, labor organizations, associations, and academia. Working through the Ministry of Health, the working group tasked a 22-member steering committee to lead the technical activities of the health benefits revision process. This study assessed the capacity of all members of the 22-member steering committee.

### Survey administration

The survey tool was pretested for clarity with five technical working group members not part of the 22 local steering-committee members. These five members were working group participants based outside Zambia. In the second round, the web-based version of the tool was administered to the same pilot sample, and their feedback was incorporated into the final revised web-based survey. Microsoft Google Forms was used as the platform for administering the online survey.

### Survey preparation

The survey information were discussed with the 22-member steering committee and the broader technical working group during a virtual benefits package meeting organized by the Ministry of Health. The participants were informed of the study’s voluntary nature, and informed consent forms were shared and discussed. Once shared, the participant had up to 1 month to complete the web-based survey. The participants were followed up during the entire survey period. The respondents were required to enter their names and institutions to eliminate multiple survey responses.

### Ethical considerations

Section one of the web-based survey contained the informed consent form, which allowed the participants to agree to continue with the survey or terminate the study at any point. The study observed anonymity and confidentiality at all stages. Ethics clearance was obtained from ERES Converge in Zambia (Apr-2022-007) and the Humanities and Social Science Research Ethics Committee at the University of KwaZulu Natal in South Africa (REC00004520/2022).

### Statistical analysis

The study applied descriptive analysis using Microsoft Excel for the participant information section. We used the R software to represent the Likert response sections graphically. No data were missing, and no additional statistical tests, including sensitivity analysis, were performed.

## Results

### Respondent characteristics

As shown in Table 1, most study participants were in the age group of 41 and 50. There were more males (68.2 percent) than females (31.8 percent). All the respondents had research proficiency in English, with only two having French as an additional language proficiency. Eighty-two per cent of the members had a master’s degree, with only nine percent with an undergraduate degree as their highest level of education. Economics and public health both equally shared 60 percent of the highest level of professional expertise. The majority of the members, 36.4 percent, had more than 20 years of experience, with only 9.1 percent having less than 5 years. Most of the committee members, at 54.5 percent, represented policymakers, followed by principal investigators from research institutions at 27.3 percent, and purchasers and payers each constituted 9.1 percent.

**Table 1.** Characteristics of the respondents and research experience

Characteristics	Frequency	Percentage
Participant age		
Less than 30	1	4.5%
31–40	7	31.8%
41–50	13	59.1%
Older than 60 years	1	4.5%
Participant sex		
Female	7	31.8%
Male	15	68.2%
International language capacity		
French	2	8.3%
English	22	100.0%
Highest level of education		
Bachelors	2	9%
Masters	18	82%
Medical doctor	2	9%
Main professional area of expertise		
Financial management	1	5%
Human sciences	1	5%
Information management	1	5%
Mental health	1	5%
Planning	1	5%
Socio-economic development	1	5%
Health financing	2	10%
Economics	6	30%
Public health	6	30%
Professional experience (in years)		
Less than 5	2	9.1%
6–10	6	27.3%
11–15	2	9.1%
16–20	4	18.2%
More than 20	8	36.4%
Type of stakeholder		
Payer of third-party healthcare services (health insurance)	2	9.1%
Policy maker (MoH HQ, MoF, other decision makers)	12	54.5%
Principle investigators of healthcare research (University/research organization)	6	27.3%
Purchaser of healthcare services (planners, hospital admin., ZAMMSA)	2	9.1%
Served on selection and/or reimbursement of health technologies committee		
No	14	63.6%
Yes	8	36.4%
Experience in conducting research		
No, 0 years	2	9.1%
Yes, 0–3 years	3	13.6%
Yes, 3–5 years	4	18.2%
Yes, 5–10 years	6	27.3%
Yes, > 10 years	7	31.8%
Use of research in everyday		
I am a research user/evidence-based practitioner	9	40.9%
I use research once in a while	8	36.4%
I work as a researcher	5	22.7%
Undertaken a systematic review or another type of evidence synthesis		
No	4	18.2%
Yes, 1–5	12	54.5%
Yes, 6–10	4	18.2%
Yes, more than 10	2	9.1%
Used the results of an HTA		
No	10	45.5%
Yes	12	54.5%
Undertaken an economic evaluation		
No	11	50.0%
Yes, 1–5	11	50.0%
Used the results of an economic evaluation?		
No	8	36.4%
Yes	14	63.6%
Critically appraised the quality of a randomized controlled trial		
No	12	54.5%
Yes	10	45.5%
Critically appraised the quality of a nonrandomized control trial		
No	16	72.7%
Yes	6	27.3%
Critically appraised the quality of an observational study		
No	13	59.1%
Yes	9	40.9%
Critically appraised the quality of a diagnostic/prognostic study		
No	15	68.2%
Yes	7	31.8%
Critically appraised the quality of a qualitative study		
No	7	31.8%
Yes	15	68.2%
Critically appraised the quality of a systematic review		
No	7	31.8%
Yes	15	68.2%
Critically appraised the quality of an economic evaluations		
No	11	50.0%
Yes	11	50.0%
Critically appraised the quality of a clinical practice guideline		
No	14	77.8%
Yes	8	44.4%

### Selection and reimbursement of health technologies committee

The majority of the steering committee, 63.6 percent, had yet to serve on any committee involved with selecting and reimbursing health technologies (Table 1). Of the eight that had served on a committee, five had served on the National Health Insurance benefits package committee; two were on the Zambia Medicines and Medical Supplies Agency committee, and one was on a Ministry of Health laboratory technologies technical working committee. Five of the eight had served on these committees between 1 and 3 years, one served between 3 and 5 years, and the remaining two served between 5 and 10 years.

### Experience in research

Most respondents (31.8 percent) had more than 10 years of research experience, followed by 27.3 percent with expertise ranging from 6 to 10 years. A total of 9.1 percent of the participants had yet to conduct any research. Most members, 40.9 percent, described themselves as research users; 36.4 percent used research evidence occasionally, whereas 22.7 percent worked as evidence producers.

### Health economics and HTA research experience

As shown in Table 1, across all the respondents, only 9.1 percent had more than 10 years of experience undertaking systematic reviews, whereas the majority (54.5 percent) had between 1 and 5 years of experience. In addition, only 54.5 percent of the members had used results from an HTA to inform decision-making. Furthermore, the experience in conducting economic evaluation was evenly split between having no expertise and having experience ranging from 1 to 5 years. The use of economic evaluation output was high at 63.6 percent. Qualitative studies and systematic reviews ranked highest at 68.2 percent in terms of expertise in different studies. The respondents had the least experience in nonrandomized trials (27.3 percent) and diagnostic studies (68.2 percent).

### Core skills

The core skills included clinical effectiveness, health economics, ethics, and patient participation.

#### Clinical effectiveness

##### HTA planning

**Figure 1.**
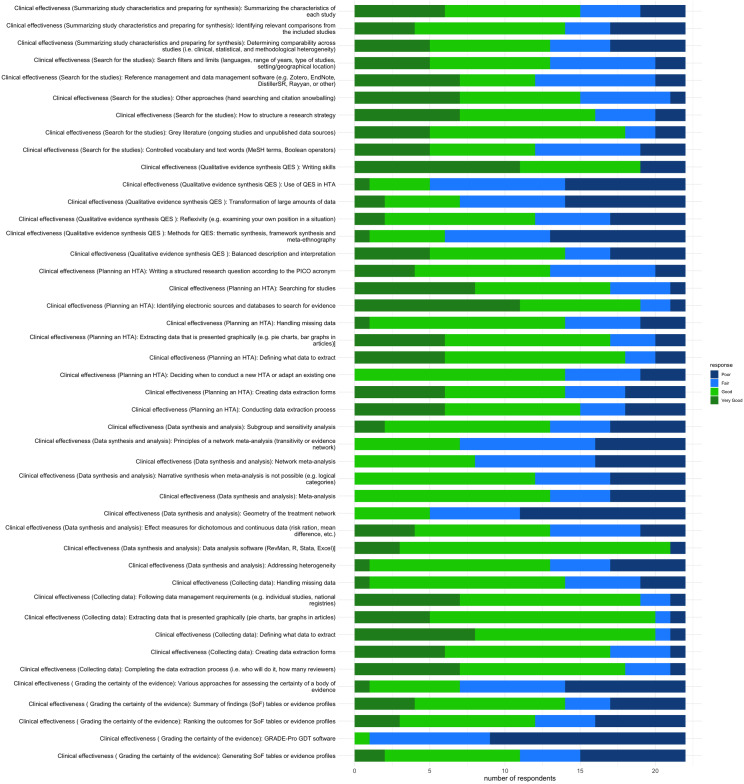
Clinical effectiveness skills.

As seen in Figure 1, when planning an HTA, respondents’ confidence levels were highest in identifying electronic sources and databases to search for evidence and lowest in structuring the research question according to the Population, Intervention, Comparison, and Outcome framework. In addition, the respondents were only slightly confident in deciding when to conduct a new HTA or adapt to an existing HTA.

##### Searching for studies

The respondents were slightly confident in searching for studies in gray literature and unpublished sources. However, they were less optimistic about structuring a search strategy and finding alternative search approaches, such as hand searches, snowballing, and using search filters. Most respondents needed more confidence using reference management software, and many needed help structuring medical subject heading terms and Boolean operators.

##### Data collection for HTA

As shown in Figure 1, most members were slightly confident in collecting the data for HTA. They had expertise in defining what data to extract from studies, creating data extraction forms, aligning these forms to national standards, and extracting data presented graphically. The respondents were least confident in handling and managing missing data.

##### Summarizing study characteristics

Concerning summarizing study characteristics and preparing literature for synthesis, 68 percent of the respondents were slightly confident and expert at summarizing the characteristics of each study. However, approximately 41 percent of the respondents only heard about approaches to determine comparable methods across studies or had no confidence in conducting them themselves.

#### Data synthesis and analysis

As seen in Figure 1 on data synthesis and analysis, respondents were slightly confident in using data analysis software such as R and STATA, meta-analysis and addressing heterogeneity, effect measures for dichotomous or continuous data, and narrative synthesis. The respondents had the least confidence in analyzing the geometry of the treatment network.

##### Qualitative evidence synthesis

More generally, more respondents were not confident enough to conduct qualitative evidence synthesis (QES). Then, 77 percent and 68 percent of the respondents did not know how to use QES to support HTA and the associated methods, such as thematic analysis and framework synthesis. Additionally, 68 percent of the respondents were equally not aware of methods to synthesize extensive qualitative data or did not have the skills to do so. The respondents were most confident in their writing skills at a mastery level.

##### Grading of certainty of evidence

Many more respondents, 64 percent, could grade evidence using the summary of findings table, and slightly more could rank the outcomes. However, 68 percent of the respondents needed skills in the various approaches to determining the certainty of evidence, and 95 percent were less confident about using the GRADE_PRO software or had never heard about it.

#### Public and patient involvement

More respondents did not have public or patient involvement skills, including those needed to identify and recruit those affected by a health technology decision, develop an engagement model, and use patient and public input.

#### Ethics

A large number of respondents, 68 percent, were aware of ethical issues with health technologies. However, 64 percent could not use models for moral philosophy, such as utilitarianism and models of justice. Half of the respondents were confident in applying bioethical issues and concepts such as informed consent and privacy, whereas the other half were not.

#### Health economics

Across all health economics skills, respondents had more confidence in conducting budget impact analysis, measuring the cost of interventions through micro-costing, and political economy analysis. The respondents had the least confidence in measuring health utility and health-related quality of life. Slightly more respondents had skills in economic evaluation, formal policy analysis, and measuring the preferences of health workers or patients via discrete choice experiments or contingent evaluation methods. Others had slightly more confidence in measuring health equity, such as estimating the incidence of catastrophic health expenditure and benefit incidence analysis. Additionally, other respondents had somewhat more confidence in measuring the economic burden of the cost of illness or macroeconomic modeling. However, respondents’ confidence was split on decision analytic modeling using decision trees, Markov models, discrete simulations, and the efficiency of the health system measurement.

### Soft skills

**Figure 2.**
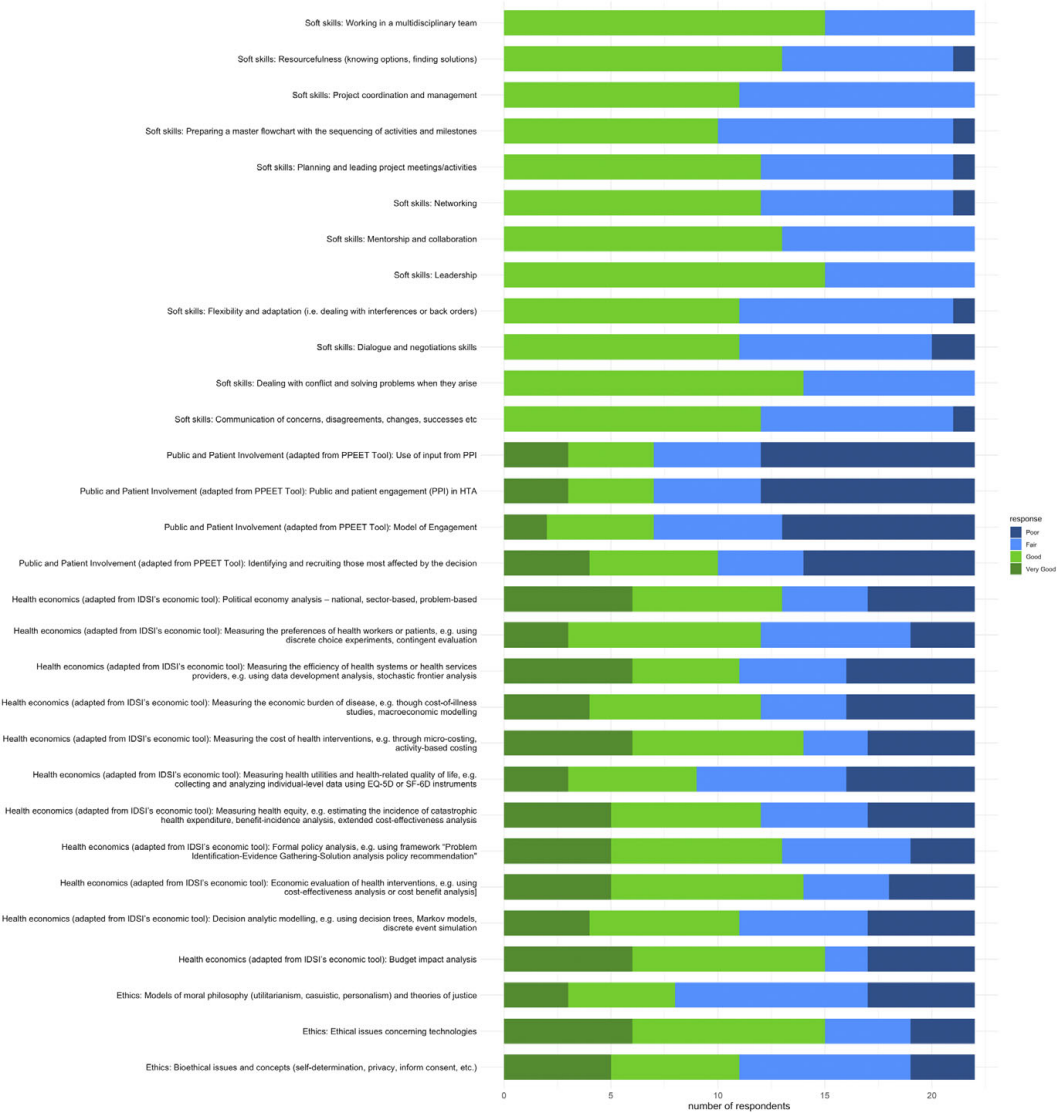
Soft, ethical, patient and public involvement, and health economics skills.

As shown in Figure 2, many more participants felt they had middle-level soft skills in leadership, working within a multidisciplinary team, networking, and dealing with conflicts. The respondents experienced less confidence in preparing the master flowchart, sequencing activities and alignments to milestones, project coordination, skills in managing negotiations, and dealing with back orders or interferences.

### Future needs

**Figure 3.**
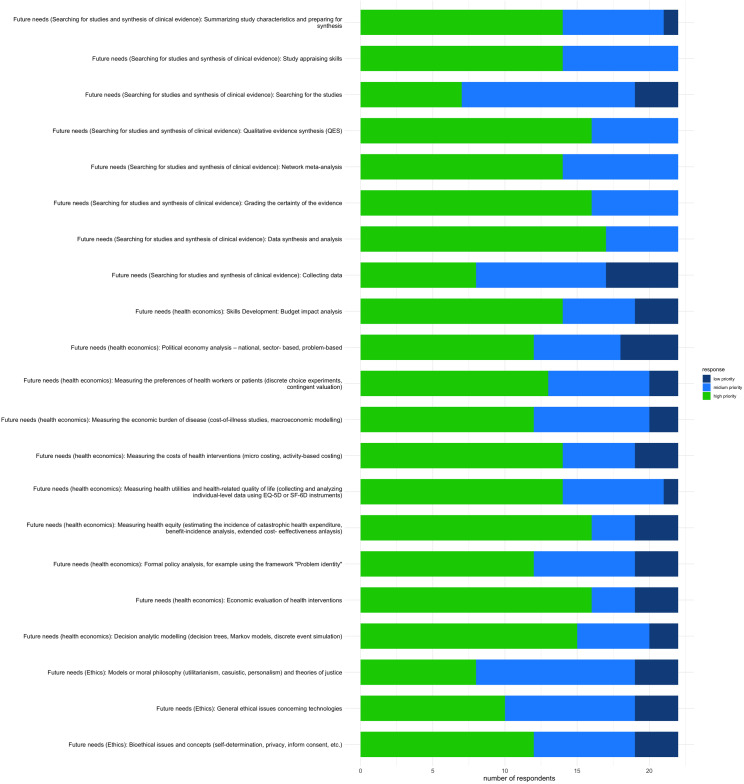
Future HTA technical capacity needs.

Figure 3 shows the skills that the respondents will seek in the future. Capacity development in health economics ranked highest. The skills sought-after included measuring health equity, economic evaluation of interventions, decision analytic modeling, budget impact analysis, utilities and health-related quality of life, and estimating the cost of health interventions. Additional high-priority skills identified were QES and data analysis of clinical evidence. Measuring the preferences of healthcare workers and patients was recognized as a future need at 59 percent. Bioethical issues and concepts, political economy analysis, and measuring the economic burden of disease were all perceived as skill development priorities by 55 percent of the participants.

## Discussion

The evidence from this study reveals high postgraduate education and experience in economics and public health, as well as a much older cohort of HTA experts. Few respondents had experience with membership in a technology selection and reimbursement committee. Most respondents had more than 10 years of experience conducting and consuming research. However, economic evaluation research expertise was limited. When planning an HTA, respondents had only slight confidence in adapting an existing HTA or conducting a new HTA. There is less capacity to structure a search strategy than to perform a search in the gray literature. While the respondents expressed some confidence in structuring the extraction forms, they were less confident handling missing data. Concerning analysis, there was more capacity in statistical analysis than skills for QES. For soft skills, the respondents had less capacity for public and patient involvement and ethics than leadership and teamwork. There was a slight confidence in health economics skills, including costing and budget impact analysis. Skills in economic evaluation, such as decision analytic modeling and health-related quality of life, were lacking.

As in similar studies, most respondents in this study have yet to serve on any health technology reimbursement committee (2;10). This could be attributed to the limited availability of committees with HTA functionalities in Zambia (22). Only a few of the respondents reported needing to conduct research. Zambia has developed deliberate policies in health research, including establishing Zambia National Health Research, to prioritize using evidence in policy decisions.

Our study evidence has shown challenges in using the Population, Intervention, Comparison, and Outcome framework in planning for HTA. Recent evidence from systematic reviews has also demonstrated that most economic evaluation studies produced in LMICs do not explicitly use the framework (2;10). Furthermore, this study revealed skills challenges in handling missing data. A global review of economic evaluation evidence also revealed increased methodological challenges in handling missing data (25). Although formal education in health economics was high, skills for its applied use were limited. In a recent examination of economic evaluation evidence to support benefits package design in Zambia, the authors recognized the absence of leadership roles in research as a significant hindrance to advancing HTA capacity in Zambia (13). In addition, the lack of a formal specialized health economics program at the available in-country academic institutions contributes to limited HTA research institutionalization (23). This is further compounded by the unavailability of an HTA government unit to support decision-making (22).

The study results also showed a need for soft skills for HTA, including project coordination. This need was also identified in other studies in LMICs on Health research programs (4;5). Like other studies, our study has demonstrated capacity gaps for patient and public involvement skills (2;4). There have been deliberate efforts to increase the quality of patient and public involvement in HTA, with notable initiatives, including the HTAi and ISPOR good practice guidelines and the evidence-informed deliberative processes framework (12;15).

The capacity to produce and review outputs of HTA at all stages facilitates the design process of the benefits package, enabling the generation, synthesis, and prioritization of evidence. In Zambia, the evidence phase of the benefits package roadmap encompasses objectives similar to various components of HTA, including the assessment and appraisal (20). In Zambia, our study has demonstrated that the requisite skills corresponding to these roadmap phases, such as preference elicitation, defining appraisal criteria, conducting and synthesizing clinical effectiveness and economic evaluations, decision analytic modeling, measuring health equity and quality of life, and performing technical efficiency analyses, require strengthening in practical application. In contrast, our study has also shown the availability of applied local skills in costing and budget impact analysis. Other countries in sub-Saharan Africa, including Ethiopia, South Africa, and Malawi, have used HTA processes to support the revision of benefits package (26–28). In Ethiopia, aspects of HTA such as scoping, stakeholder engagement, generation of preference criteria for prioritization, synthesis of cost-effectiveness evidence, costing of intervention unit costs, and budget impact analysis have been utilized to support the formulation of its essential health benefits package (28). South Africa established a short-term HTA technical working group to promote capacity development for creating and implementing the National Health Insurance benefits package (27). In designing the health benefits package for 2023 to 2030, Malawi synthesized cost-effectiveness evidence and employed multi-criterion decision analysis utilizing societal preferences alongside resource needs estimates to facilitate prioritization (26).

The policy implications of these results first underscore the need for HTA capacity development initiatives to design and implement the National Health Care Package effectively. The evidence further highlights the networking gaps needed by external institutions with the capacity of HTA to support its strengthening in Zambia. In addition, as most skills involve high knowledge levels but less practical expertise, there is a need for deliberate policy to engage stakeholders in HTA production and active use in Zambia. These policy implications are significant now in Zambia, as it has positioned HTA as a critical instrument in its health financing strategy and health benefits package reform. Recently, stakeholders rejected the amendments to the national health benefits packages due to insufficient evidence-based deliberation (29). As a result, policymakers must ensure adequate capacity for designing and revising benefits packages that yield acceptable outcomes.

### Limitations

Although the initial survey tool discussion with respondents and follow-up maximized the response rate and potentially improved the response data quality, the web-based and self-administered nature of the survey was susceptible to socially desirable responses. Additionally, the study only reviewed the HTA capacity within the technical steering committee established by the Ministry of Health for benefits package revision, which is not an exhaustive list of all possible HTA capacities in Zambia. Consequently, the generalizability of the study results must be applied with caution.

## Conclusion

This study has curated Zambia’s HTA capacity, which is available to support revising and implementing the national health benefits package. The evidence calls for increased technical capacity strengthening in health economics, clinical effectiveness, ethics, patient and public involvement, and soft skills to support HTA production and use. To accomplish this, promoting local leadership roles in HTA research, enhancing the transferability of capacity for HTA research within the country, fostering strategic partnerships with global HTA agencies, identifying and mapping local opportunities for applying HTA principles, and establishing a formal HTA institution domestically will be essential.

## Supporting information

Simangolwa et al. supplementary material Simangolwa et al. supplementary material

## Data Availability

All data relevant to the study are included in the article or uploaded as supplementary information.

## Supplementary material

The supplementary material for this article can be found at http://doi.org/10.1017/S0266462325000030.

## Patient and public involvement

Patients and the public were not involved in this research’s design, conduct, reporting, or dissemination.
